# Origin of High-Rate Performance of Sb_2_Se_3_/Sb Composite Anodes for Na-Ion Batteries

**DOI:** 10.1021/acsaem.5c02418

**Published:** 2025-09-30

**Authors:** Amalie Skurtveit, Faduma M. Maddar, Ivana Hasa, Carmen Cavallo, David S. Wragg, Alexey Y. Koposov

**Affiliations:** † Centre for Materials Science and Nanotechnology, Department of Chemistry, 6305 University of Oslo, PO Box 1033, Blindern, 0315 Oslo, Norway; ‡ WMG, The University of Warwick, Coventry CV4 7AL, United Kingdom; § Department of Battery Technology, Institute for Energy Technology (IFE), Instituttveien 18, 2007 Kjeller, Norway

**Keywords:** Na-ion batteries, anode materials, *operando* XRD, SEM-EDX, mechanism, high-rate cycling

## Abstract

Improving the energy density of Na-ion batteries (NIBs) requires advances in anode materials. This study investigates Sb_2_Se_3_/Sb composite anodes, which offer better capacity and stability than the individual constituents. *Operando* X-ray diffraction (XRD) revealed that (de)­sodiation is driven by synergies between the operating mechanisms of Sb_2_Se_3_ and Sb, enabling a reversible capacity of 360 mAh g^–1^ (at 1000 mA g^–1^). This performance is also aided by amorphization of the sodiated phases, which promotes rapid Na-diffusion. The work emphasizes the need for continuous material and structural optimizations to fully harness their benefits for enabling efficient and fast-charging NIBs.

## Graphical abstract

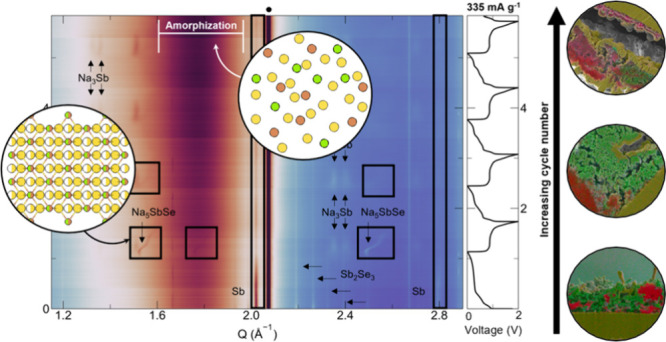

Na-ion batteries (NIBs) gained initial attention as a potential low-cost solution for stationary energy storage applications and have been recently accepted into the transport sector.^1–3^ Despite the greater atomic weight of Na compared to Li (22.94 g mol^–1^ vs. 6.94 g mol^–1^), which affects energy density, its larger ionic radius allows for the development of high-power batteries.^4^ This is due to the low surface-charge density, which reduces the interaction with Lewis bases (e.g., electrolyte solvents and salts).^4^ Consequently, NIBs offer potential advantages in higher power densities compared with those of Li-ion batteries (LIBs). However, a set of materials capable of handling high current densities is quite limited, and a deeper understanding of their chemistry must be achieved to enhance their response in terms of lifetime and storage capacity at high rates. Historically, advancements in cathode materials’ chemistry have outpaced those of anode materials,^5^ leaving a gap in knowledge that must be addressed. Practical materials for NIB anodes are mainly limited to hard carbons,^6^ the performance of which at high current densities can be achieved at the cost of capacity loss. Thus, improvements in power density characteristics of NIBs can be achievable through advancements in anode chemistry, particularly through the exploration of new anode materials.

Recent research demonstrated a promising performance of conversion/alloying anode materials in NIBs^7^ under high cycling rates (up to 1000 mA g^–1^ for Sb_2_Se_3_),^8–11^ indicating their relevance for applications demanding high power capabilities. The development of these materials was driven by the poor cycling performance of alloying materials, which often suffer from degradation associated with the volumetric expansion.^12^ The conversion/alloying reaction of Sb_2_Se_3_ anodes proceeds through several steps, including the formation of Sb nanoparticles embedded in a Na–Se matrix (conversion reaction), which provides a network beneficial for electron and Na-ion transport; Sb further alloys with Na to form Na_3_Sb.^13^ More recently, we have demonstrated that Na_3_Sb (amorphous) and Na_2_Se (nanocrystalline) react during the sodiation process, resulting in a new ternary Na_5–*x*_SbSe phase as the final sodiated product.^14^ This set of reactions allows 12 Na-ions to be stored per formula unit of Sb_2_Se_3_, leading to a high theoretical capacity of 670 mAh g^–1^. However, the practical access to this high capacity is limited by the irreversible conversion reaction occurring during the first sodiation. The Na_2_Se matrix formed during the conversion reaction is insulating, which can decrease the electrical conductivity of the electrode and affect the electrochemical performance at high current densities. Typically, these issues are addressed by fabrication of material blends or composite materials (e.g., Sn-hard carbon composites,^15^ reduced graphene oxide (rGO)-coated Sb_2_Se_3_, or polypyrrole-coated Sb_2_Se_3_).^11,16^ Some composite materials often include electrochemically inactive components such as Sb/MgF_2_ ^17^ responsible for stabilization of electrochemical performance. Replacing this inactive part with an active one through formation of heterostructures (SnS_2_/Mn_2_SnS_4_,^18^ Sb_2_S_3_@FeS_2_ ^19^) or composite materials with metal­(loids) (e.g., Sb_2_O_3_/Sb^20^), has been shown to be one of pathways to achieve high-capacity anodes with good electronic conductivities. The conversion reaction also negatively contributes to the first cycle Coulombic efficiency (CE) as some of the alkali-metal ions are irreversibly trapped in the matrix formed after the conversion reaction. To improve reversibility and avoid losses during the first cycle, nonstoichiometric materials (e.g., SiN_*x*_ in LIBs) were proposed.^21^ The intentional introduction of N-deficiency in SiN_*x*_ allows one to shift the balance between conversion and alloying reactions, improving the first cycle CE.^21^ Due to the nature of the conversion/alloying reaction, materials adopting this storage mechanism are expected to undergo morphological changes which can affect long-term cyclability and overall cell performance. Such morphological changes taking place during cycling include sintering-, reconstruction-, or activation-type mechanisms, and are rather unexplored.^22^ Insights into the morphological changes that take place during electrochemical cycling are crucial for the fundamental understanding of the operation mode of conversion and alloying materials and can identify potential pathways for their optimization.

In this work, we explored a Sb_2_Se_3_/Sb composite (75/25 wt % ratio) as high-capacity anodes for high-rate applications, which was evaluated using *operando* X-ray diffraction (XRD) at different current densities: 100, 335, and 1000 mA g^–1^. We also examined the morphological changes of Sb_2_Se_3_/Sb composite electrodes at several stages of cycling by ex-situ scanning electron microscopy (SEM) coupled with energy-dispersive X-ray (EDX) spectroscopy. This combined structural and morphological analysis allowed us to explore the origin of the high capacity of Sb_2_Se_3_/Sb composite anodes in NIBs at high cycling rates and to elucidate the causes of capacity fade as a consequence of the morphological evolution of the electrodes during electrochemical cycling.

Sb_2_Se_3_/Sb composite was synthesized through a solvothermal procedure similarly to the description available in the literature, with slight modifications to tune the chemical composition of the final product.^11^ The ratio between the Sb:Se-precursors was 4:2.4 mmol, yielding Sb_2_Se_3_/Sb composite with approximately 75 and 25 wt % of Sb_2_Se_3_ and Sb, respectively. This composition was determined by quantitative Rietveld analysis, which confirmed two-phase system behavior where Sb_2_Se_3_ crystallizes in the orthorhombic space group *Pbnm* and Sb crystallizes in the rhombohedral *R*3̅*m* crystal structure (Figure 1a). In this work, the *Pbnm* setting for Miller indexing of planes in Sb_2_Se_3_ was used.^23^ An impurity phase, Sb_2_O_3_ (cubic, *Fd*3̅*m*), was identified from Rietveld refinement (Figure 1a), but this phase would have a negligible effect on the electrochemical performance of the composite material. Further crystallographic information for the materials can be found in the Supporting Information (SI) (see Table S1). The SEM micrograph revealed that Sb_2_Se_3_ had a rodlike crystallite shape (∼2.5–5 μm long), while particles of Sb were evenly distributed throughout the sample and had spherical-like morphology (<0.5 μm) (see Figure 1b, as well as Figure S1).

**Figure 1:**
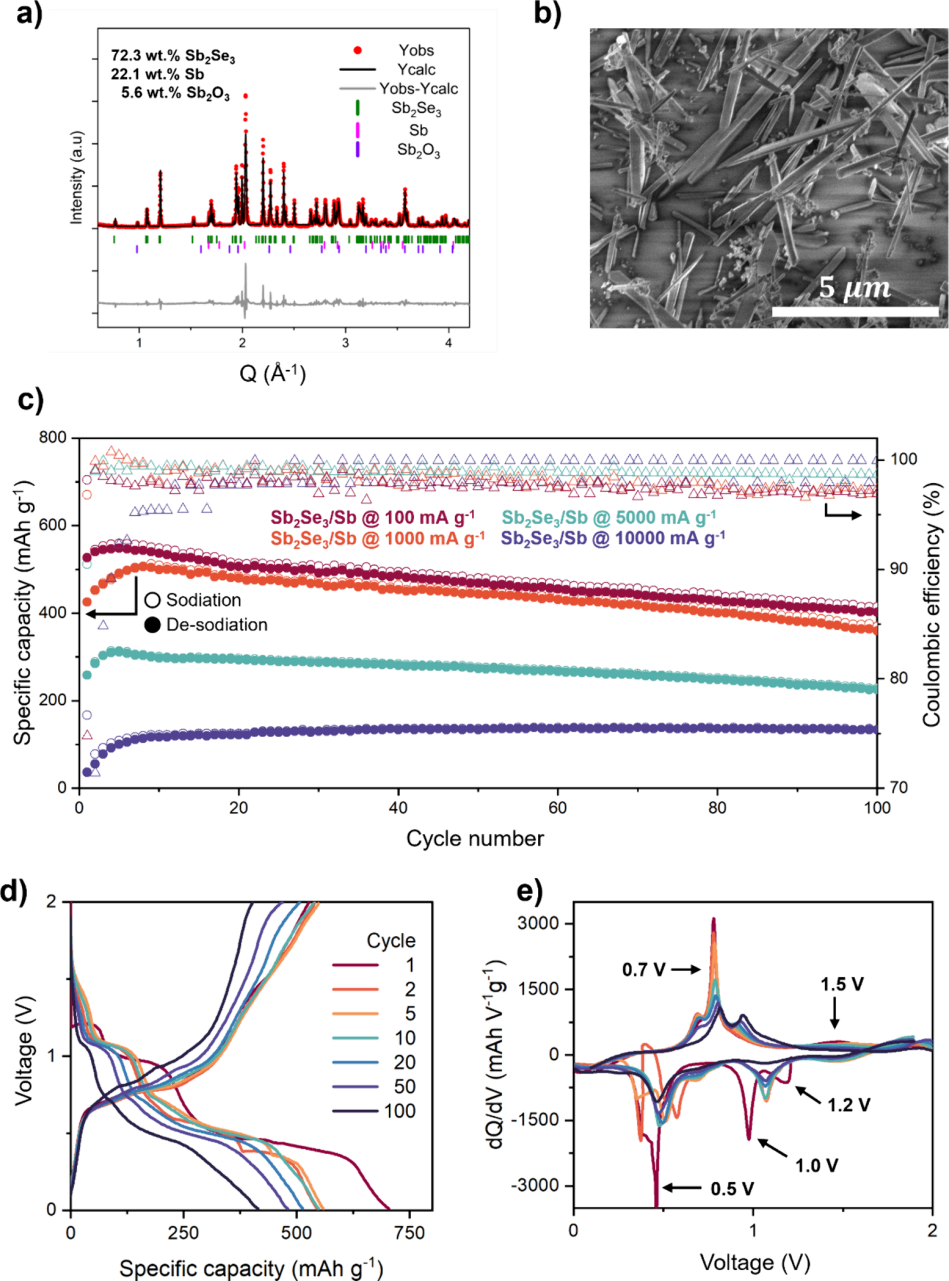
(a) Rietveld refinement fit plot for the Sb_2_Se_3_/Sb composite and (b) SEM micrograph of the pristine powder. (c) Capacity per cycle plot of Sb_2_Se_3_/Sb in Na-half cells cycled between 0.01 and 2.00 V vs Na/Na^+^ at 100, 1000, 5000, and 10000 mA g^–1^. Open and closed symbols are sodiation and desodiation, respectively. (d) Sodiation/desodiation curves for Sb_2_Se_3_/Sb anode in Na-half cell cycled between 0.01 and 2.00 V vs Na/Na^+^, using a specific current density of 100 mA g^–1^. (e) d*Q*/d*V* curves derived from the GC profiles.

The Sb_2_Se_3_/Sb-based electrodes were galvanostatically cycled at 100 mA g^–1^ (Figures 1c and 1d). The first sodiation and desodiation specific capacity of Sb_2_Se_3_/Sb was 704 and 527 mAh g^–1^, respectively, with an initial CE of 75% (see Figure 1d, Table S2). In the following cycle, the sodiation and desodiation capacity decreased to 545 and 540 mAh g^–1^. The higher capacities observed during the first cycle were attributed to the formation of a solid-electrolyte interphase (SEI) and the irreversible conversion reaction. After the expected capacity decrease during the second cycle, the capacity partly recovered in cycles 3–10, which typically is referred to as an electrochemical activation caused by the exposure of new surfaces within active material.^22^ Sb_2_Se_3_/Sb composite exhibited a relatively stable cycling stability over 100 cycles, maintaining a specific capacity of 400 mAh g^–1^ with a CE of ∼97% resulting in a capacity retention of 74%. The composition of Sb_2_Se_3_/Sb material was found to be beneficial for increasing both the initial specific sodiation capacity and the reversible capacity at the 100th cycle, compared to the parent Sb_2_Se_3_ material explored in the previous study (initial capacity and capacity at the 100th cycle of Sb_2_Se_3_: 648 and 235 mA g^–1^, respectively).^14^ The performance of the composite in a full-cell configuration was evaluated by coupling the Sb_2_Se_3_/Sb-based electrodes with Prussian White (PW) cathodes with a composition of Na_2_Fe­[Fe­(CN)_6_]·2H_2_O (Figure S2), using the same electrolyte as was used for the fabrication of half-cells (i.e., 1 M NaPF_6_ in PC + 5 vol % FEC). Herein, the capacities are reported with respect to the mass of the anode active material, and sodiation refers to sodiation of Sb_2_Se_3_/Sb. The specific sodiation capacity of the first cycle of Sb_2_Se_3_/Sb||PW was 865 mAh g^–1^ with an initial CE of 53% (Figure S2). In the following cycles, the capacity stabilized ∼330 mAh g^–1^ with a CE of ∼98% (Figure S2). After the 100th cycle, the specific sodiation capacity of Sb_2_Sb_3_/Sb||PW was 173 mAh g^–1^ with a CE of ∼98%. The first cycle energy density of the Sb_2_Se_3_/Sb||PW full cell cycled at 100 mA g^–1^ was calculated to be ∼200–300 Wh kg^–1^ (total weight of both electrodes, excluding current collectors). The full cell performance of Sb_2_Se_3_/Sb||PW was comparable to that reported for other conversion/alloying materials (Table S2).^8^

The high-rate performance of the Sb_2_Se_3_/Sb composite was systematically evaluated using specific current densities of 1000, 5000, and 10 000 mA g^–1^ (Figure 1c) based on the results obtained from rate capability tests (Figure S3). Sb_2_Se_3_/Sb composite cycled at 1000 mA g^–1^ exhibited a first specific sodiation capacity of 665 mAh g^–1^ (Figure S4), which is similar to the performance reported at 100 mA g^–1^. The GC sodiation/desodiation curves of Sb_2_Se_3_/Sb tested at 1000 mA g^–1^ resembled those measured at 100 mA g^–1^, which suggests that the cycling progressed in a similar manner, even at high rates (Figure S4). The electrochemical performance was stable for 100 cycles, maintaining a specific sodiation capacity of 360 mAh g^–1^ and CE of ∼97% at 1000 mA g^–1^. At 5000 mA g^–1^, the specific capacity decreased to ∼225 mAh g^–1^ at the 100th cycle, while at 10 000 mA g^–1^, the reversible specific sodiation capacity was ∼130 mA g^–1^ (Figure 1c, Table S2).

To understand the (de)­sodiation mechanism of the Sb_2_Se_3_/Sb composite and reveal the structure–property relationship that leads to the high-rate performance (Figure 1c), *operando* XRD was performed using specific current densities of 100, 335, and 1000 mA g^–1^ (corresponding to C-rates of ∼C/6, ∼C/2, and ∼2C, respectively, based on the theoretical capacity of Sb_2_Se_3_, 670 mAh g^–1^) (Figure S5, Figure 2). The (de)­sodiation mechanism of the Sb_2_Se_3_/Sb composite was examined through a combination of d*Q*/d*V* (Figure 1e) and *operando* XRD (Figure S5) conducted at 100 mA g^–1^. The electrochemical reaction of Sb_2_Se_3_/Sb proceeded through multiple steps as evidenced from the GC sodiation/desodiation and corresponding d*Q*/d*V* curves (Figures 1d and 1e). The first peak in the d*Q*/d*V* was located at 1.2 V vs. Na/Na^+^ during the first sodiation can be correlated to the initial intercalation of Na ions between adjacent ribbons within the crystal structure of Sb_2_Se_3_ and formation of SEI (Figure 1e).^11^ The following sodiation peak at 1.00 V vs. Na/Na^+^ corresponds to the initial conversion reaction yielding Na_2_Se and Sb (Figure 1e). From the *operando* XRD of Sb_2_Se_3_/Sb cycled at 100 mA g^–1^ (Figure S4), it is evident that Na_2_Se forms as nanocrystalline or amorphous, similar to what was reported earlier.^24^ The conversion of Sb_2_Se_3_ into Sb was observed as a broadening effect on the Sb (102̅) reflection, located ∼2 Å^–1^ (Figure S5). The last d*Q*/d*V* sodiation peak (0.49 V vs. Na/Na^+^) and its shoulder was ascribed to the alloying reaction forming Na_*x*_Sb (*x* = 1–3) phases, along with the formation of the recently discovered Na_5_SbSe (Figure 1e).^14^ The formation of Na_3_Sb (*P*6_3_/*mmc*, No. 194) and Na_5_SbSe (*I*4/*mmm*, No. 139) is manifested by the reflections located at 1.34, 1.52, 2.35, and 2.40 Å^–1^ (Na_3_Sb), and the reflections at 1.52, 1.76, and 2.48 Å^–1^ (Na_5_SbSe) in the *operando* contour plot (Figure S5).^14^

**Figure 2:**
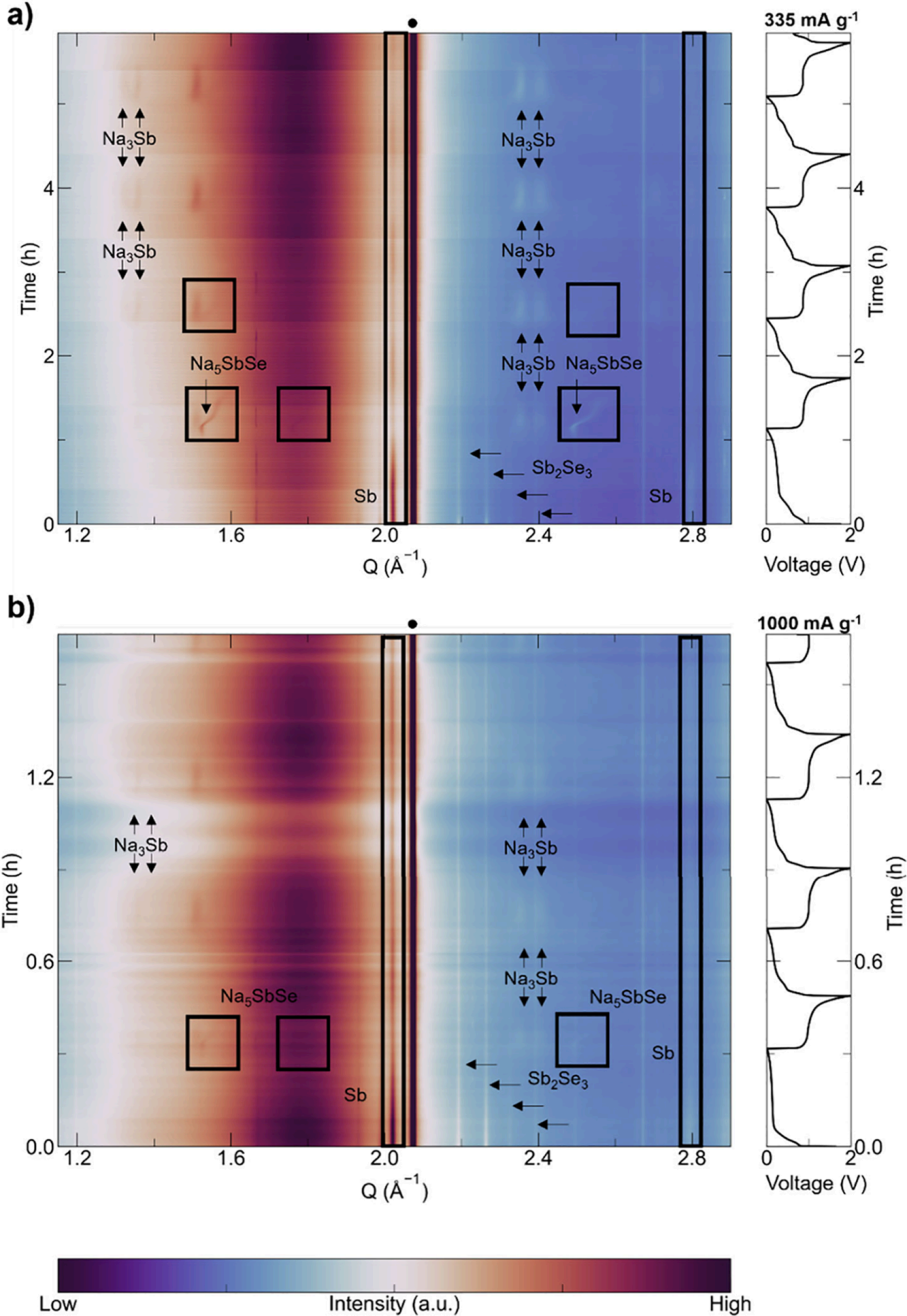
*Operando* XRD contour plots of Sb_2_Se_3_/Sb composite cycled between 0.01 and 2.00 V vs Na/Na^+^ in a half cell using a specific current density of (a) 335 mA g^–1^ and (b) 1000 mA g^–1^. Diffraction peaks of the starting phases (Sb_2_Se_3_ and Sb) and the phases appearing during cycling (Na_3_Sb and Na_5_SbSe) are marked with black rectangles or black arrows. Reflections corresponding to other inactive cell components (e.g., Na) are marked with solid black circles at the top of the contour plots.

The d*Q*/d*V* peak observed at 0.77 V vs. Na/Na^+^ during the first desodiation corresponds to the dealloying reaction of Na_3_Sb (forming Sb) and deinsertion of Na ions from the Na_5–*x*_SbSe structure (typical values of *x* ≈ 1–3) (Figure 1e).^14^ This process was observed as a shift of reflections corresponding to Na_5–*x*_SbSe to higher *Q* values, as described previously (Figure S5).^14^ A small bump in the d*Q*/d*V* curve at 1.5 V vs. Na/Na^+^ during desodiation was attributed to complete deinsertion of Na ions from the Na_5–*x*_SbSe phase (forming Sb and Na_2_Se) (Figure 1e). This process marks the end of the desodiation process, where only reflections corresponding to Sb can be observed at 2.82 Å^–1^ (Figure S5). The intercalation and conversion reactions appeared to be irreversible under these cycling conditions (i.e., 0.01–2.00 V vs. Na/Na^+^) (Figures 1d and 1e). The voltage at which the formation of Na_3_Sb and Na_5_SbSe occurs is slightly different in the second sodiation compared to the first sodiation process (Figure 1e). After this transition (2nd cycle), the consecutive cycles are reproducible, highlighting the reversibility of the reactions.

The origin of the high-rate performance of the Sb_2_Se_3_/Sb composite was explored through *operando* XRD measurements over several (de)­sodiation cycles using current densities of 335 and 1000 mA g^–1^ (Figures 2a and 2b, respectively). Such *operando* characterization can be performed only at synchrotron facilities due to short acquisition time and, therefore, high time resolution, which allows to track the subtle phase transitions occurring through high-rate cycling.^25^ Sb_2_Se_3_/Sb cycled at 335 and 1000 mA g^–1^ behaved in a similar manner to what was observed in the *operando* XRD performed at 100 mA g^–1^ during the first (de)­sodiation cycle, forming Na_3_Sb and Na_5_SbSe (Figure 2). During the second sodiation for the cell cycled at 335 mA g^–1^, the intensity of the reflections corresponding to the Na_5_SbSe phase (1.52, 1.76, and 2.48 Å^–1^) decreased substantially (Figure 2a); they completely disappeared for the cell cycled at 1000 mA g^–1^ (Figure 2b). Simultaneously with the disappearance of these reflections, the contribution of amorphous phases (which can be observed as the red area between 1.6 Å^–1^ and 1.95 Å^–1^ in Figures 2a and 2b) considerably increased, indicating that Na_5–*x*_SbSe becomes amorphous under these cycling conditions (Figure S6). It should be noted that Na_5–*x*_SbSe typically maintains its crystallinity even at higher cycle numbers when cycled at 100 mA g^–1^.^14^ Such amorphization of Na_5–*x*_SbSe indicates that fast diffusion of Na-ions can be achieved in such structures similarly to what was observed for amorphous Na_*x*_Sb (*x* = 1–3).^13^ NIBs are generally associated with “sluggish” sodiation kinetics due to the larger ionic radius and higher mass of Na than Li.^26^ The fast-charging results of Sb_2_Se_3_/Sb contradict the view on sluggish kinetics of sodiation; some materials can enable rapid extraction/insertion of Na ions while still following complete (de)­sodiation pathways, even at high rates.

Morphological changes of the active materials are expected to occur for conversion and alloying materials and known to influence their electrochemical performance.^27^ Therefore, Sb_2_Se_3_/Sb-based electrodes were extracted after the 1st, 5th, 10th, 20th, and 120th (de)­sodiation cycles and evaluated using SEM-EDX (see Figure 3, as well as Figures S7–S14). The analysis of the micrographs revealed substantial morphological changes when comparing the surface and cross sections of the electrodes extracted after the first and 120th desodiation (see Figure 3, as well as Figure S7).

**Figure 3:**
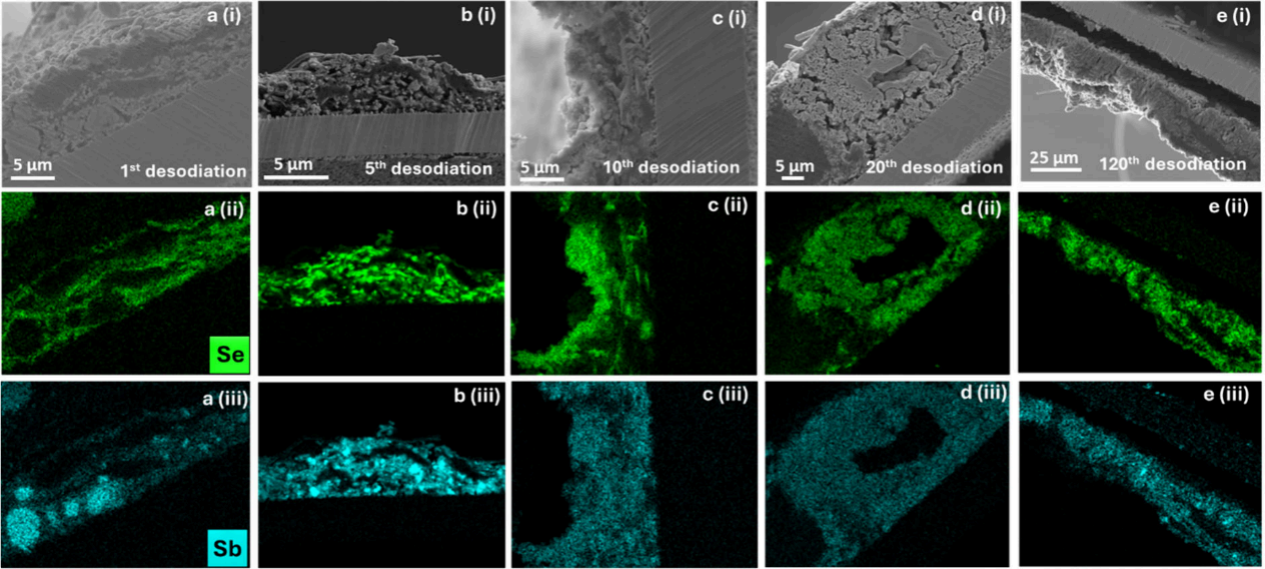
Row (i) shows cross-sectional micrographs of a Sb_2_Se_3_/Sb electrode after the (a) 1st, (b) 5th, (c) 10th, (d) 20th, and (e) 120th desodiation, with corresponding Se and Sb EDX maps in rows (ii) and (iii), respectively.

The rod-like morphology of Sb_2_Se_3_ (as displayed in Figure 1b) is preserved during the first cycle, as highlighted in the electrode cross-section of the first desodiation (2.00 V vs. Na/Na^+^) (Figures 3a­(i) and 3a­(ii)). However, as the cycling progressed, the defined rodlike morphology agglomerated into small clusters by the fifth cycle (Figures 3b­(i) and 3b­(ii)). The SEM-EDX micrographs of the electrodes extracted after the sodiation process (1st, 5th, 10th, 20th, and 120th) indicated a good elemental overlap between Na, Sb, and Se, which is further attributed to the formation of Na_3_Sb, Na_2_Se, and Na_5_SbSe (see panels marked “(a)” in Figures S9–S14) and supports the findings from the *operando* XRD described above (see Figure 2, as well as Figure S5). The SEM-EDX micrographs of the Sb_2_Se_3_/Sb electrodes extracted after the first and fifth desodiation showed that primary particles of Sb with a size of ∼2–5 μm agglomerated within the electrode (see Figures 3a­(iii) and 3c). This observation is further supported by *operando* XRD, where the reflections corresponding to Sb become quite distinct (indicating the growth of the crystalline fragments) at this point of cycling (Figure 2). Such agglomeration is often encountered for alloying-type elements and has been observed in similar studies.^28^ Often, the mobility, agglomeration, and volume expansion/contraction of Sb (∼390% from Sb to Na_3_Sb)^29^ and other alloying elements during the (de)­sodiation process are the main cause for electrode failure.^28^ However, the observed agglomerates of Sb seemingly redisperse across the electrode in the 10–120th (de)­sodiation cycles, as shown in the EDX maps (Figure 3c–e)). Note that this is not the main cause of electrode failure in the Sb_2_Se_3_/Sb composite system. Even though the material delivered a specific sodiation capacity of ∼400 mAh g^–1^ at the 100th cycle (74% capacity retention), the electrochemical performance degraded quickly between the 120th and 150th cycles (Figure S15). Similar performance of Sb_2_Se_3_/Sb was also observed when the material was coated on carbon-coated Al foil: the slight differences in electrochemical behavior are attributed to different interaction of active material with the current collector (Figure S16). Electrodes extracted after the 120th desodiation cycle revealed that the surface morphology exhibited a mossy-like structure and that the active part of the electrode had cracked (Figure S14). Moreover, cross-sectional SEM-EDX analysis enabled an understanding of the observed rapid capacity fading of Sb_2_Se_3_/Sb, and the micrographs showed that the electrode coating had delaminated from the Cu current collector, also highlighting regions of Sb phase separation and agglomeration (see Figure 3e, as well as Figures S13 and S14).

In summary, the Sb_2_Se_3_/Sb composite (75/25 wt %) demonstrated a considerable potential to enhance the practical capacity of Sb_2_Se_3_ anodes at high rates while mitigating the volume expansion/contraction problems associated with the use of pure alloying materials such as Sb. The composite was tested using multiple current densities and demonstrated sodiation capacities of 400, 360, 225, and 133 mAh g^–1^ at 100, 1000, 5000, and 10000 mA g^–1^, respectively (after 100 cycles). *Operando* XRD revealed that the (de)­sodiation mechanism of the Sb_2_Se_3_/Sb composite progresses through a combination of the reaction mechanisms of Sb_2_Se_3_ and Sb, forming Na_3_Sb and Na_5_SbSe at the end of sodiation. *Operando* XRD conducted at high rates (335 and 1000 mA g^–1^) confirmed that the performance was driven by the amorphization of compositionally flexible Na_5–*x*_SbSe, facilitating rapid Na diffusion due to the high configurational disorder inherent in this phase. Cross-sectional SEM-EDX imaging identified delamination of the active part from the Cu current collector as the primary cause of capacity degradation after 120 cycles. With further improvements such as optimization of the electrode formulation, electrolyte compatibility studies, and presodiation strategies, Sb_2_Se_3_/Sb composites promise to be viable NIB anodes for high-power applications.

## Supplementary Material



## Notes

Experimental section, electrochemical data (including full-cell, high-rate, and rate capability tests), *operando* XRD of Sb_2_Se_3_/Sb at 100 mA g^–1^, micrographs, EDX maps (PDF)

## ∇

For C. Cavallo: FAAM, Fib spa, Strada Statale Via appia 7 bis – 81030, Teverola (CE), Italy

## ABBREVIATIONS

NIBs: Na-ion batteries
LIBs: Li-ion batteries
XRD: X-ray diffraction
CE: Coulombic efficiency
GC: galvanostatic cycling
SEM: scanning electron microscopy
EDX: energy-dispersive X-ray spectroscopy
