# Supplementary material for: Origin of High-Rate Performance of Sb2Se3/Sb Composite Anodes for Na-Ion Batteries
Source: ACS Appl Energy Mater. 2025 Sep 30;8(19):14058–64. doi: 10.1021/acsaem.5c02418 (PMC12522094; doi:10.1021/acsaem.5c02418)
Supplement: Supplementary file 1 [file ae5c02418_si_001.pdf]

Supporting Information for "Origin of High-Rate Performance of Sb_2Se_3/Sb Composite Anodes for Na-ion Batteries"

Amalie Skurtveit[1], Faduma M. Maddar[2], Ivana Hasa[2], Carmen Cavallo[1†], David S. Wragg[1,3], Alexey Y. Koposov[1]**

[1]Centre for Materials Science and Nanotechnology, Department of Chemistry, University of Oslo, PO Box 1033, Blindern, 0315 Oslo, Norway

[2]WMG, The University of Warwick, Coventry, CV4 7AL United Kingdom

[3]Department of Battery Technology, Institute for Energy Technology (IFE), Instituttveien 18, 2007 Kjeller, Norway

[†]Current address: FAAM, Fib spa, Strada Statale Via appia 7 bis – 81030, Teverola (CE), Italy

*Correspondence to amalie.skurtveit@kjemi.uio.no and alexey.koposov@kjemi.uio.no

Experimental section

$SbCl_3$ (≥ 99.0 %), Se (≥ 99.5 %), $NaBH_4$ (≥ 98.0 %), Na metal (dry), propylene carbonate (PC, 99.7 %), fluoroethylene carbonate (FEC, anhydrous, ≥ 99 %), carboxymethylcellulose sodium salt (CMC, high-viscosity) and citric acid (99.5 %) were purchased from Sigma-Aldrich. KOH (99.9 %) was purchased from Merck. Super P was purchased from Timical. Ethylene glycol (≥ 98.0 %) and ethanol (99.5 %) were purchased from VWR and Antibac, respectively. $NaPF_6$ was purchased from Fluorochem. Prussian white (PW) cathode material, with a chemical composition of $Na_2Fe[Fe(CN)_6]*2H_2O$ was provided by Altris AB. Na, PC, FEC, and $NaPF_6$ were stored inside an Ar-filled glovebox. MBraun glovebox (H_2O and O_2 levels below 0.1 ppm) was used for all the work conducted in an inert Argon (Ar) atmosphere. Two-sided dendritic Cu foil (99.9 %) was purchased from Schlenk. Single carbon coated Al foil was purchased from Cambridge Energy Solutions. All chemicals were of reagent grade and used without further purification.

The synthesis of the Sb_2Se_3/Sb composite material was slightly modified from a procedure previously reported in literature.[1, 2] In a typical synthesis, $SbCl_3$ (4 mmol, 0.9125 g) was dissolved in ethylene glycol (60 mL) under continuous stirring. In a separate beaker, Se (2.4 mmol, 0.1924 g) and $NaBH_4$ (8 mmol, 0.3024 g) were reacted in ethanol (10 mL). The resulting Se-precursor solution was rapidly added dropwise to the Sb-precursor solution under vigorous stirring resulting in the formation of a black precipitate. After stirring for ~5 min, the reaction mixture was transferred to three Teflon-lined autoclaves (~20 mL volume each), sealed and heated at 200 °C for 12 h. The grey powder (~0.5 g) was isolated by centrifugation (5 min at 5000 rpm) and washed three times with ethanol (3x35 mL). The powder was dried under dynamic vacuum overnight at 60 °C.

Sb_2Se_3/Sb was characterized using X-ray diffraction (XRD) and scanning electron microscopy (SEM). For the XRD characterization, a Bruker D8 Discover diffractometer equipped with a LynxEye detector and monochromatic CuKα1 radiation with a wavelength of 1.540596 Å was used. The morphology of pristine Sb_2Se_3/Sb was studied with a high-resolution Hitachi SU8230 cold-field emission scanning electrode microscope, generating micrographs using secondary electrons. The sample was placed on top of carbon tape to prevent movement.

Electrodes based on Sb_2Se_3/Sb were prepared in a similar manner to our previous work, using a 6:2:2 ratio of Sb_2Se_3/Sb, Super P, and CMC in distilled water and a KOH/citric acid buffer (pH between 2 and 3).[3] The resulting slurry was coated on a two-sided dendritic Cu foil (10 μm) using an applicator with a gap of 200 μm and dried overnight in air. Electrodes (Ø15 mm) were cut and dried in dynamic vacuum in a Büchi oven for 4 h at 80 °C, before they were transferred to an Ar-filled glovebox (MBraun Labmaster, H_2O and O_2 levels below 0.1 ppm). The active mass loadings of the electrodes were ~1.2-1.5 mg per electrode (~0.68-0.85 mg/cm^2). A Sb_2Se_3/Sb-based slurry, by using with the same composition and procedure as above, was coated on a carbon coated Al-foil (16 μm), in order to compare the electrochemical results between coating on Cu and Al-foil. Electrodes comprised of the PW cathode material were prepared similarly to previous work,[3] using a 8.5:1:0.5 ratio between

PW, Super P, and CMC. The slurry was casted on a carbon coated Al-foil using an applicator gap of 500 μm. The cathode electrode sheet was dried overnight, before electrodes (Ø15 mm) were cut and dried in a Büchi oven at 140 ˚C for ~12 h and transferred to an Ar-filled glovebox. The active mass loadings of the electrodes were ~6-7 mg per electrode (~3.40-3.96 mg/cm^2).

The electrochemical behavior of Sb_2Se_3/Sb was assessed in CR2032 coin cells (Pi-kem or Neware, 304 steel type). All cells were made inside an Ar-filled glovebox. Na metal disks were used as the counter electrode, while glass fiber (Whatman GF/C, Ø16 mm) was used as a separator. The electrolyte was 1 M $NaPF_6$ in PC + 5 v.% FEC, which was prepared in the glovebox by dissolving $NaPF_6$ salt (1.6814 g) in PC (9.5 mL) and FEC (0.5 mL). The Na disks were prepared immediately prior to cell assembly by cutting off a fresh piece from Na blocks with a scalpel, then rolling it out to a thin sheet and cutting out Ø14 mm disks (~80 mg). The surfaces of Na disks were brushed with a toothbrush. All cells were crimped using a hydraulic TMAXCN coin cell crimper. The electrochemical characterization was carried out at 25 ˚C using a potential range between 0.01 V and 2.00 V *vs.* Na/Na^+ for the half cells. Galvanostatic cycling (GC) was performed using a Neware battery tester (CT-4008T-5V10mA-164 and MIHW-200-160CH, Neware) using specific current densities of 100 mA g^{-1}, 1000 mA g^{-1}, 5000 mA g^{-1} and 10 000 mA g^{-1} without any formation cycles. GC rate capability testing was performed using current densities of 100, 335, 1000, 5000 mA g^{-1} (4 cycles at each current density), before the current went back to 100 mA g^{-1} (10 cycles). The full cells were cycled between 1.3 V and 3.8 V *vs.* Na/Na^+ using the same electrolyte.

Operando XRD measurements were performed at the BM01 and BM31 beamlines at the Swiss-Norwegian Beamlines (SNBL), European Synchrotron Radiation Facility (ESRF), Grenoble, France. The cell assembly of all *operando* cells was similar to the process described above, but a customized cell equipped with X-ray transparent windows (glassy carbon) was used.[4] BM01 is equipped with a Dectris Pilatus 2M area pixel-count detector and a wavelength of 0.60546 Å was used, while BM31 is equipped with a Dectris Pilatus3X 2M CdTe area-pixel detector and a wavelength of 0.31916 Å was used. The 2D diffractograms were azimuthally integrated to yield 1D diffraction patterns using Bubble.[5] The *operando* dataset collected at the BM31 beamline has been corrected for nonuniform response in the pixels of the detector (gain correction) and integrated with Python scripts provided by the BM31 staff.[6] The cells were cycled galvanostatically at current densities of 100, 335, and 1000 mA g^{-1} using a Biologic MPG2 battery cycler (for the cell measured at BM31, specifically the cell cycled at 100 mA g^{-1}), and Bat-Small battery cycler (Astrol) (for the cells measured at BM01, specifically the cells cycled at 335 and 1000 mA g^{-1}) between 0.01 V and 2.00 V *vs.* Na/Na^+. Attempts of cycling the Sb_2Se_3/Sb composite at 5000 mA g^{-1} in *operando* mode were made, but the electrochemical cell used for *operando* XRD measurements did not allow to cycle at such high current density. The *operando* XRD contour plots were prepared using Python v. 3.7, and the script is available as a GitHub repository.[7]

Electrodes were retrieved in sodiated and de-sodiated state from coin cells after cycling to characterize them using SEM coupled with energy-dispersive X-ray (EDX) spectroscopy. All coin cells were cycled between 0.01 V and 2.00 V *vs.* Na/Na^+ with a specific current density of 100 mA g^{-1}

using a Neware battery tester (MIHW-200-160CH). The performance of the cells was compared to the baseline performance to ensure a representative selection of cells. The cells were opened using a Hohsen de-crimper inside an Ar-filled glovebox. SEM-EDX imaging was performed on a Zeiss Sigma microscope. The Sb_2Se_3/Sb-based electrodes were transferred from the glovebox into the microscope using an airless transfer system. Analysis was performed by applying an acceleration voltage of 10 kV. Cross-sections of the electrodes were obtained using a HITACHI Ion Milling system (IM4000) using an acceleration voltage of 4.5 V for 2 hours with a swing of ± 40°.

Supporting tables and figures

Table S1. Crystallographic information of the crystal structures used for Rietveld refinements and analysis in this work. Values are extracted from crystallographic information files (CIFs) obtained from the crystallographic open database (COD) and Cambridge Crystallographic Data Centre (CCDC).

Phase	Space group (No.)	Z	Unit cell parameters (Å)	Atom	Wyckoff	Atomic sites *x*	*y*	*z*	Source
Sb_2Se_3	*Pbnm* (62)	4	*a* = 11.620	Sb1	4c	0.3280	0.03050	0.2500	9007437 (COD)
			b = 11.77	Sb2	4c	0.0397	0.14780	0.7500	
			c = 3.962	Se1	4c	0.8732	0.05340	0.2500	
				Se2	4c	0.4434	0.13020	0.7500	
				Se3	4c	0.19350	0.21320	0.2500	
Sb	*R*-3*c* (167)	6	*a* = 4.300	Sb1	6c	0.000	0.0000	0.23362	2310879 (COD)
			c = 11.222						
Na_5SbSe	*I*4/*mmm* (139)		*a* = 5.050	Na1	2b	0	0	0.5	2433581 (CCDC)
			c = 7.169	Na2	4d	0	0.5	0.25	
				Sb1	2a	0	0	0	
				Se1	2a	0	0	0	
Na_2Se	*Fm*-3*m* (225)	2	*a* = 6.8230	Se1	4a	0.0000	0.0000	0.0000	1538738 (COD)
				Na1	8c	0.2500	0.25000	0.2500	
Na_3Sb	*P*6$_3$/*mmc* (194)	2	*a* = 5.3550	Sb1	2c	1/3	2/3	0.2500	1010292 (COD)
			c = 9.4960	Na1	2b	0.000	0.000	0.0000	
				Na2	4f	1/3	2/3	0.5830	
Na	*Im*-3*m* (229)	2	*a* = 4.2820	Na1	2a	0.0000	0.0000	0.0000	8104233 (COD)
Cu	*Fm*-3*m* (225)	4	*a* = 3.6154	Cu1	4a	0.000	0.0000	0.0000	4105681 (COD)

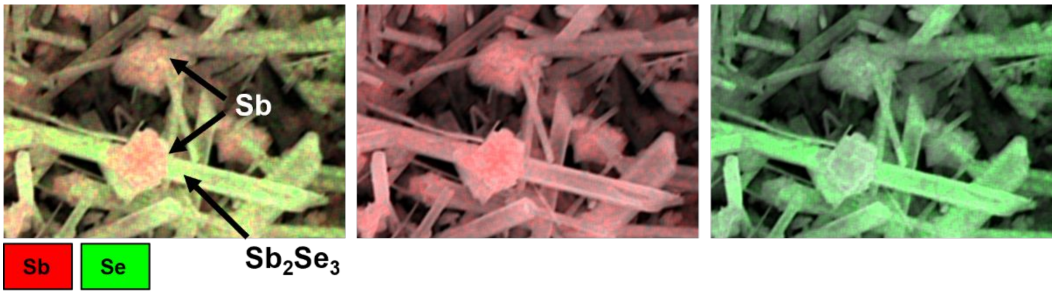

Figure S1. EDX mapping of the pristine Sb_2Se_3/Sb composite material displaying the rod-like crystallite shape of Sb_2Se_3 and spherical-like Sb particles.

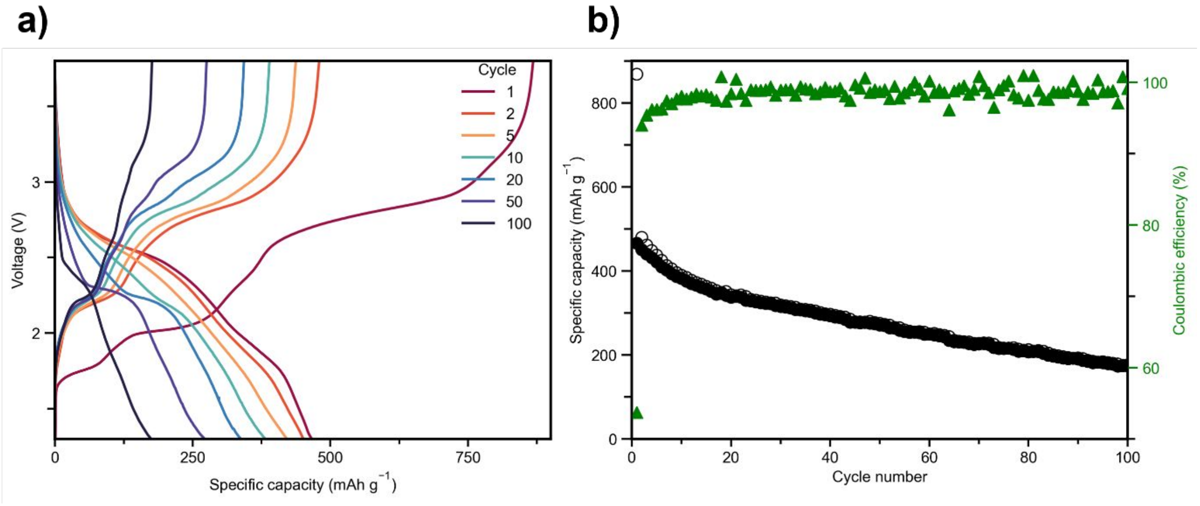

Figure S2. a) Galvanostatic sodiation-de-sodiation curves of Sb_2Se_3/Sb||Prussian white (PW) full cell cycled between 1.3 V and 3.8 V using a specific current density of 100 mA g^{-1} (with respect to the anode material). **b)** Specific capacity upon cycling and Coulombic efficiency trend of the Sb_2Se_3||PW full cell. Open and closed symbols represent sodiation and de-sodiation, respectively. Note that the specific capacities are represented in respect to the anode mass for direct comparison with half-cell performances.

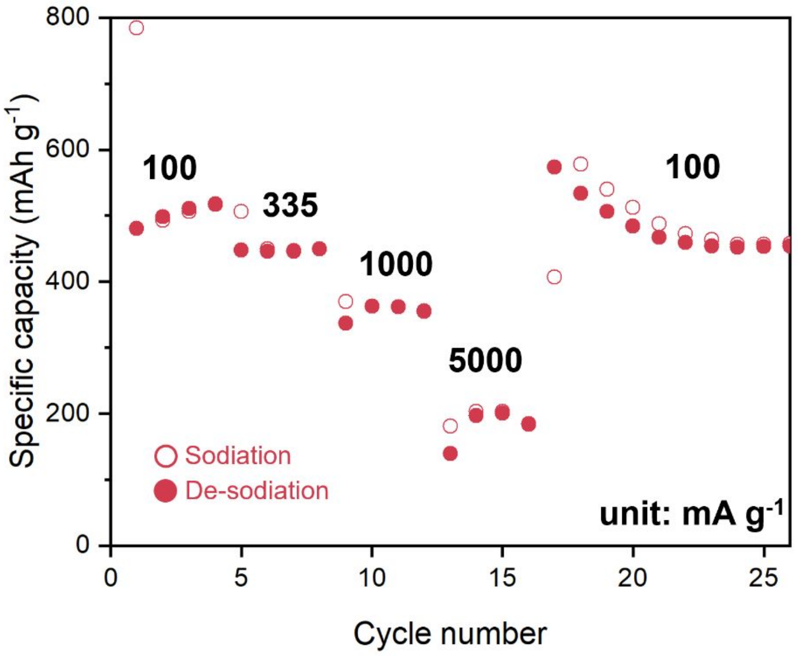

Figure S3. GC rate capability test of Sb_2Se_3/Sb composite at selected current densities of 100 mA g^{-1}, 335 mA g^{-1}, 1000 mA g^{-1} and 5000 mA g^{-1} (C-rate: ~C/6, ~C/2, ~2C, and ~8C, respectively). Open and closed symbols are sodiation and de-sodiation, respectively. The capacity increases as the cycling progresses for each current density, indicating that the material slowly adjusts to the current. When the current is switched e.g., from 100 to 335 mA g^{-1} after the 4th cycle, the sodiation capacity is higher than the de-sodiation capacity (open and closed symbols) suggesting a large overpotential. When the current is reverted back to 100 mA g^{-1}, the initial sodiation capacity is restored after the material had adjusted to the current.

Table S2. Electrochemical performance comparison between this work and other conversion/alloying materials as anodes in NIBs.

Material (active material content, %)	Electrolyte	Specific current density (mA g^{-1})	Reversible capacity (mAh g^{-1}) (Cycle No.)	Potential window (V)	ICE (%)	Reference
Sb_2Se_3/Sb (60)	1 M $NaPF_6$ in PC + 5 % FEC	100	400 (100)	0.01-2.00	75	This work
Sb_2Se_3/Sb (60)	1 M $NaPF_6$ in PC + 5 % FEC	1000	360 (100)	0.01-2.00	63	This work
Sb_2Se_3/Sb (60)	1 M $NaPF_6$ in PC + 5 % FEC	5000	225 (100)	0.01-2.00	50	This work
Sb_2Se_3/Sb (60)	1 M $NaPF_6$ in PC + 5 % FEC	10000	133 (100)	0.01-2.00	22	This work
Sb_2Se_3/Sb‖PW (85)	1 M $NaPF_6$ in PC + 5 % FEC	100	200 (100)*	1.3-3.8	93	This work
s-Sb_2Se_3 (60)	1 M $NaPF_6$ in PC + 5 % FEC	100	235.4 (100)	0.01-2.0	59	[3]
Sb_2Se_3/rGO (70)	1 M $NaCF_3SO_3$ in DEGCME	100	~580 (100)	0.01-3.0	72.6	[1]
Sb_2MoO_6 (80)	1 M $NaClO_4$ in PC:EC (1:1) + 5 % FEC	1000	498.7 (100)	0.01-3.0	n.a.	[8]
Sb_2MoO_6‖NVP/C (1:7 ratio)	1 M $NaClO_4$ in PC:EC (1:1) + 5 % FEC	200	571.9 (1)*	2.5-3.8	98**	[8]
Bi_2Te_3/PPy (60)	1 M $NaClO_4$ in PC:EC (1:1) + 5 % FEC	1000	~350 (GC-rate)	0-2.5	n.a.	[9]
Large Sb_2S_3 NPs (n.a.)	1 M $NaClO_4$ in PC + 3 % FEC	2400	~600 (100)	0.01-2.5	~97	[10]

* Reported with respect to the mass of anode material, ** 5 formation cycles of the cathode material before assembling full cells

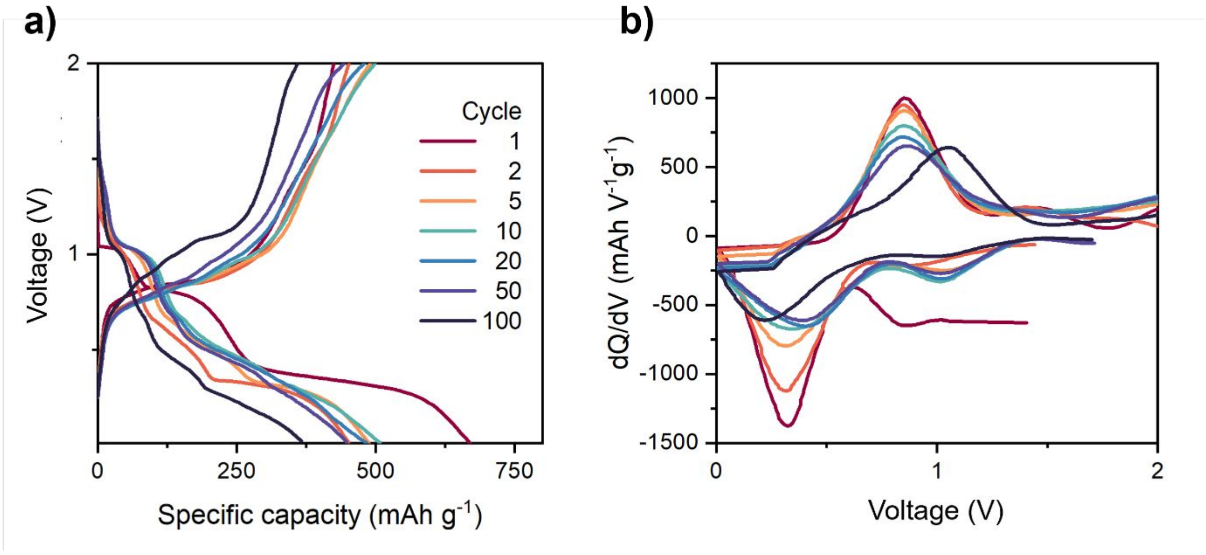

Figure S4. **a)** GC sodiation-de-sodiation curves of Sb_2Se_3/Sb cycled at 1000 mA g^{-1} in Na-half cell between 0.01 V and 2.00 V, and **b)** corresponding dQ/dV traces. Extracted from the same cell as displayed in Figure 1c in Main. The GC traces resemble that of the traces measured at 100 mA g^{-1} (see Figure 1d in Main), albeit a higher overpotential can clearly be observed for the traces at 1000 mA g^{-1} indicated by the larger shift of the redox couples in **b)**.

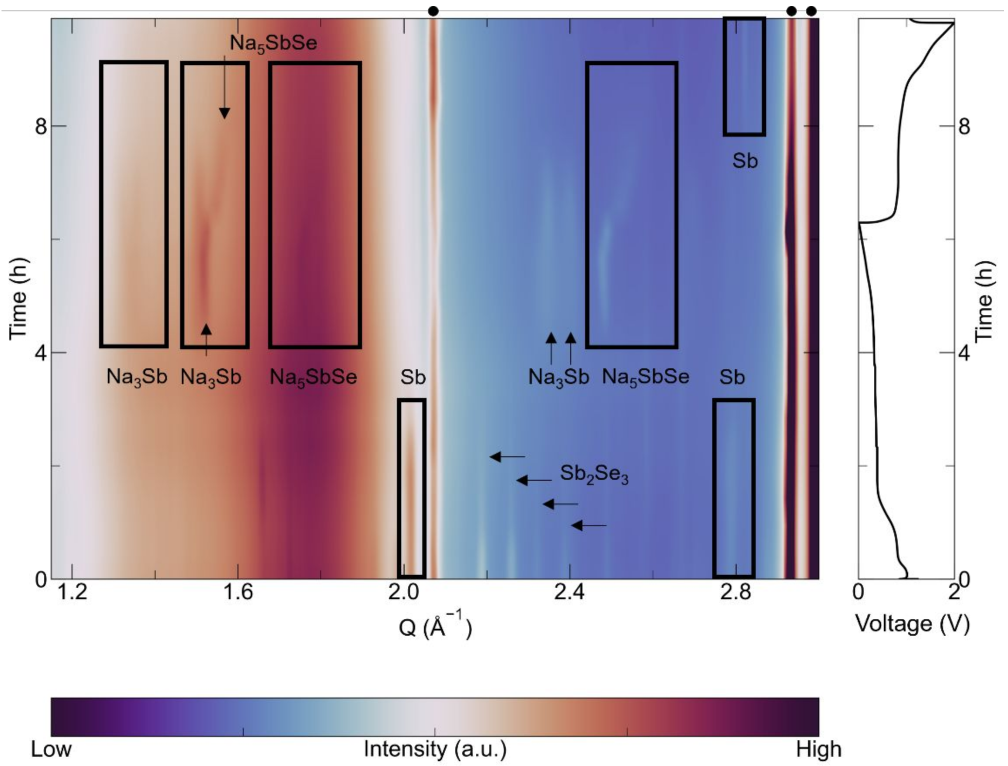

Figure S5. *Operando* XRD contour plot of Sb_2Se_3/Sb composite cycled between 0.01 V and 2.00 V *vs.* Na/Na^+ in half cell using a specific current density of 100 mA g^{-1}. Diffraction peaks of the starting phases (Sb_2Se_3 and Sb) are marked with black rectangles and arrows. Phases appearing during cycling (Na_3Sb and Na_5SbSe) are represented with black rectangles. Reflections corresponding to other inactive cell components (e.g., Na and Cu) are marked with black solid circles at the top of the contour plot.

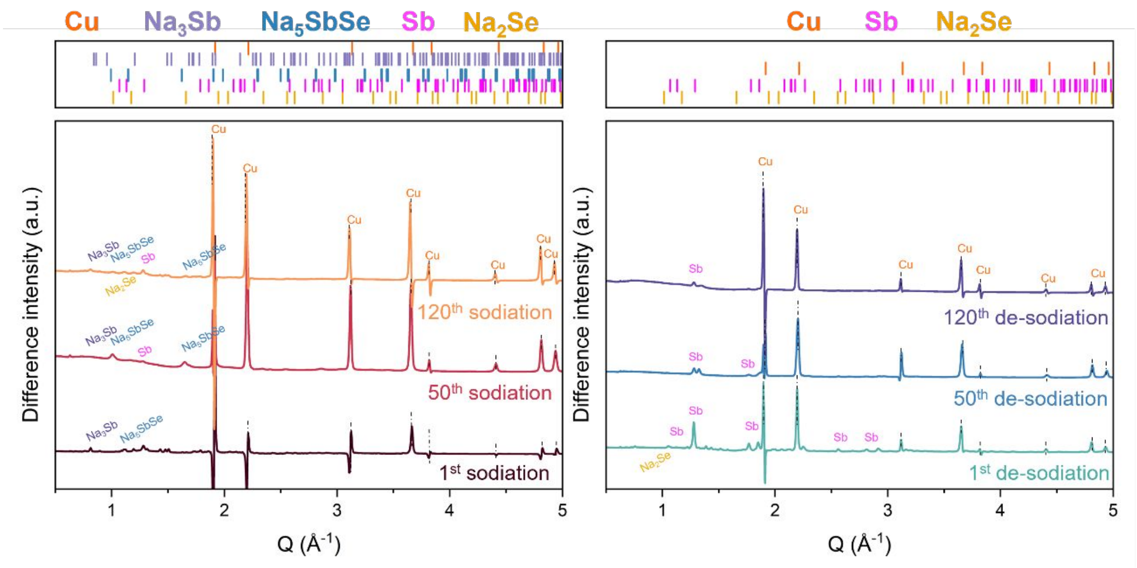

Figure S6. XRD patterns of Sb_2Se_3/Sb electrodes extracted after the 1st, 50th, and 120th (de-)sodiation. The cells were cycled at 1000 mA g^{-1} using Na metal as the counter electrode. All patterns have been normalized and subtracted the normalized intensity of pristine Cu-foil (difference plots), which allows to observe weaker reflections corresponding to the phases appearing during the (de-)sodiation process (e.g., Na_3Sb, Na_5SbSe (and isostructural $Na_{5-x}SbSe$), Sb, and Na_2Se, whose Bragg positions are displayed at the top of the plots).

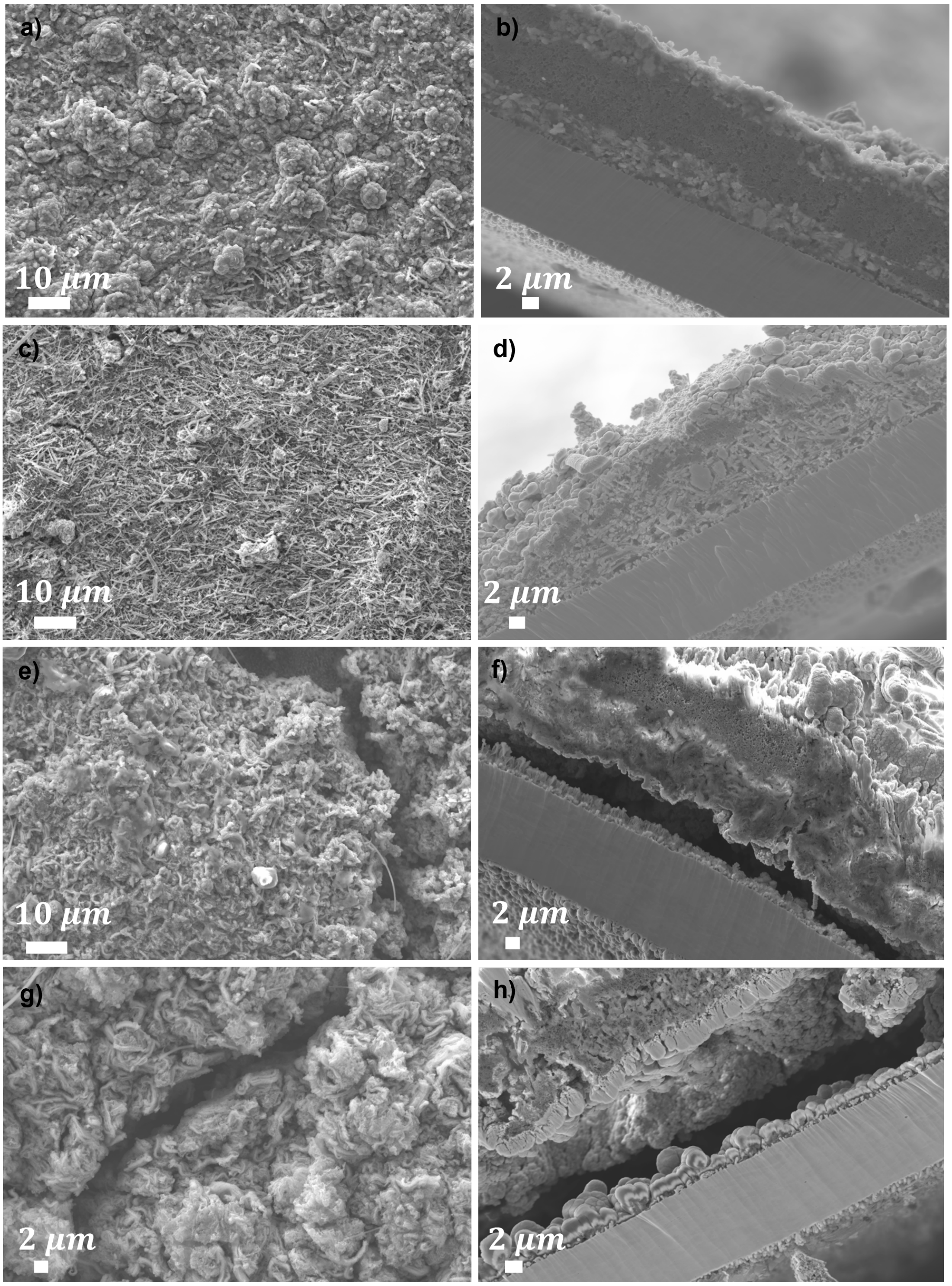

Figure S7. SEM micrographs of cycled Sb_2Se_3/Sb electrodes at key stages of de-sodiation. **a-b)** 1st sodiation, **c-d)** 1st de-sodiation, **e-f)** 120th sodiation, and **g-h)** 120th de-sodiation. Surface of the electrodes are shown on the left, while the cross-section of the same electrodes is shown on the right.

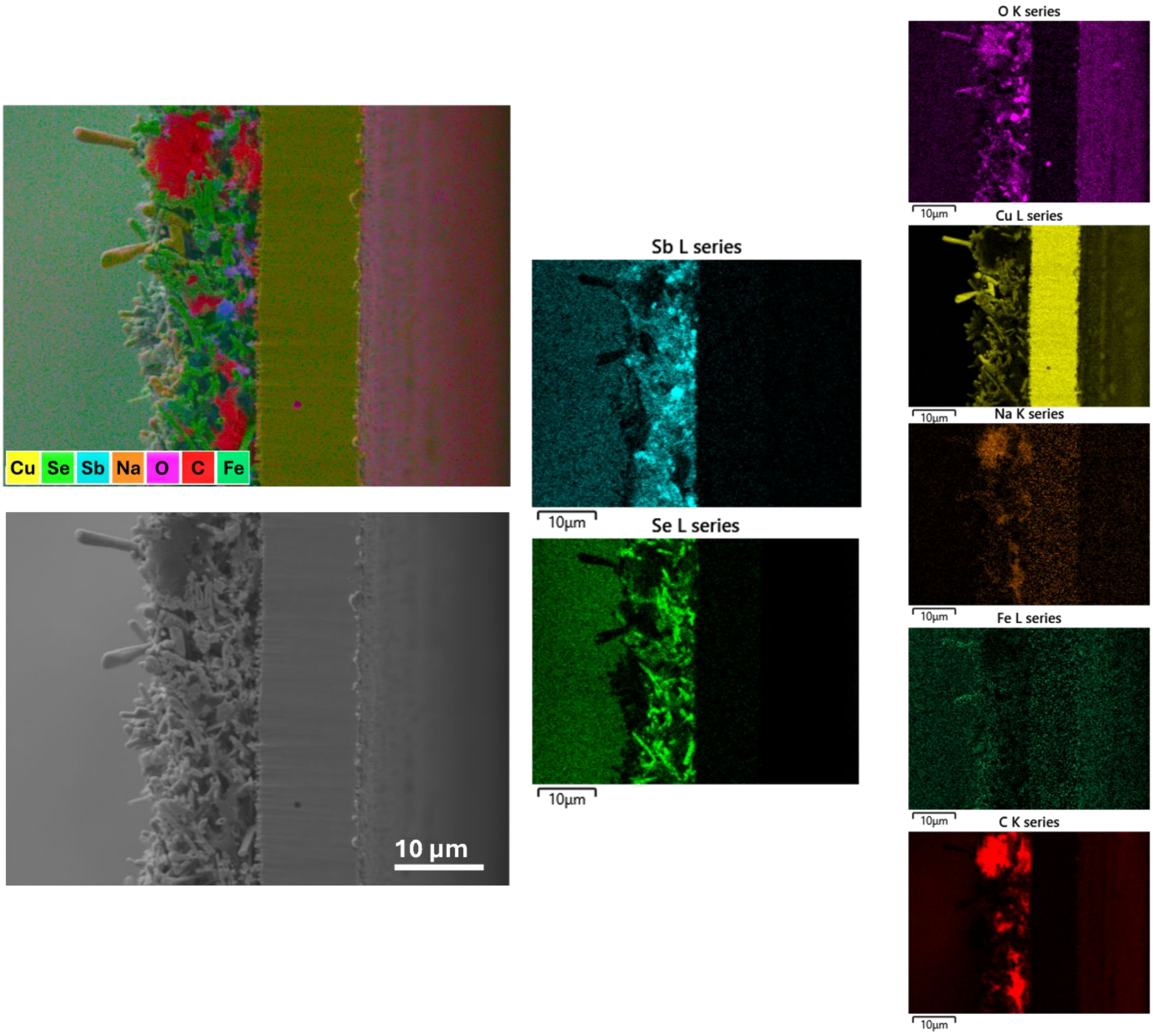

Figure S8. Cross-sectional EDX mapping of the pristine electrode based on the Sb_2Se_3/Sb composite. The EDX map showed a good elemental overlap between Sb and Se and showed that the active material is homogeneously dispersed throughout the electrode. Carbon (Super P) and binder agglomeration is observed as indicated with red color in the C map.

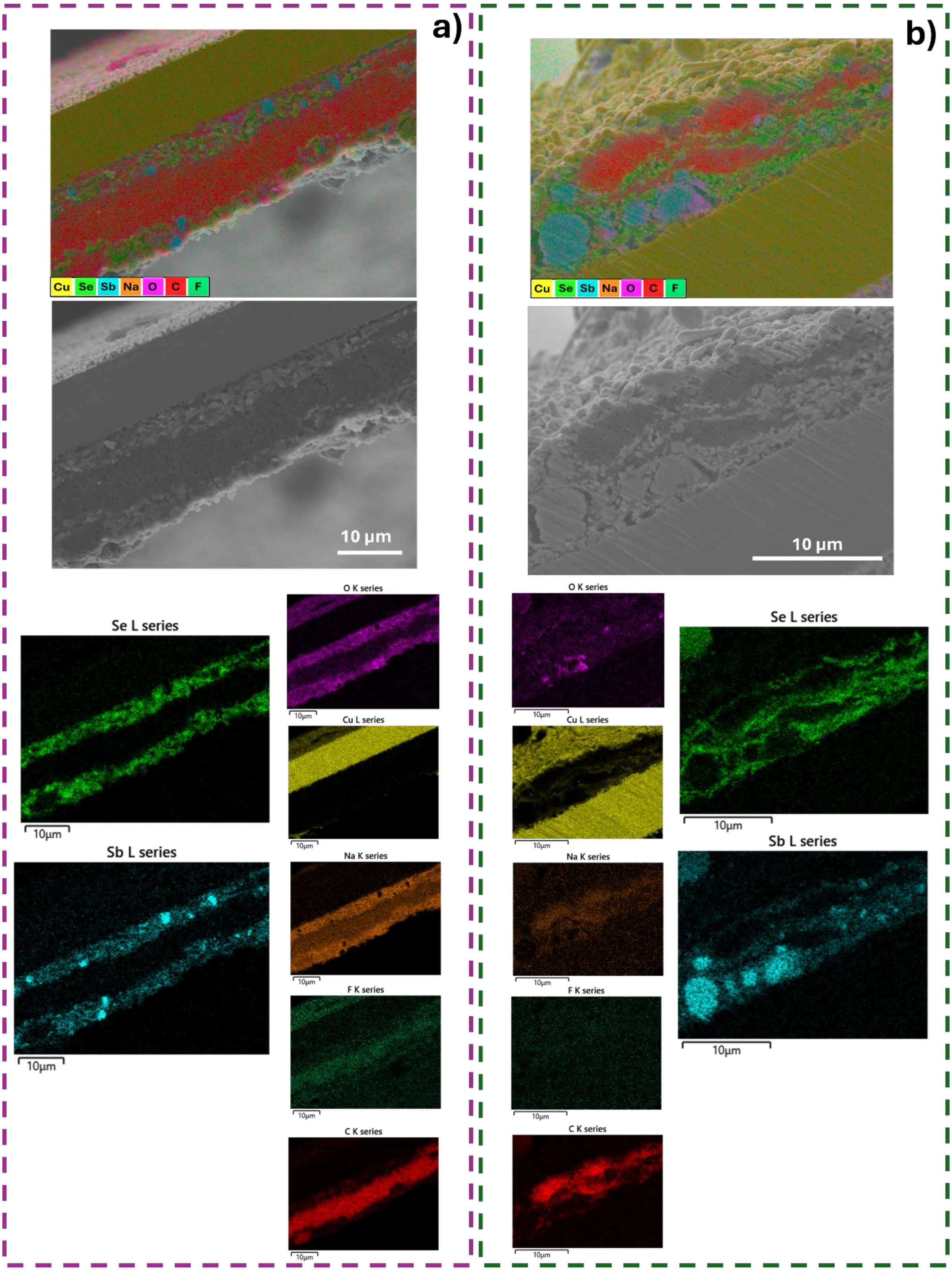

Figure S9. Cross-sectional EDX mapping of Sb_2Se_3/Sb-based electrodes retrieved after **a) (purple dashed line)** the 1st sodiation, and **b) (green dashed line)** the 1st de-sodiation. The EDX mapping of the 1st sodiation in **a)** showed that the whole electrode was sodiated (orange color in map) and that phases corresponding to Na_3Sb (cyan color) and Na_2Se (green color) and Na_5SbSe (cyan and green colors) could be observed. The EDX mapping of the 1st de-sodiation in **b)** showed that elemental Sb (cyan color) is formed (2.00 V) and Na_2Se remained a stable compound (green color).

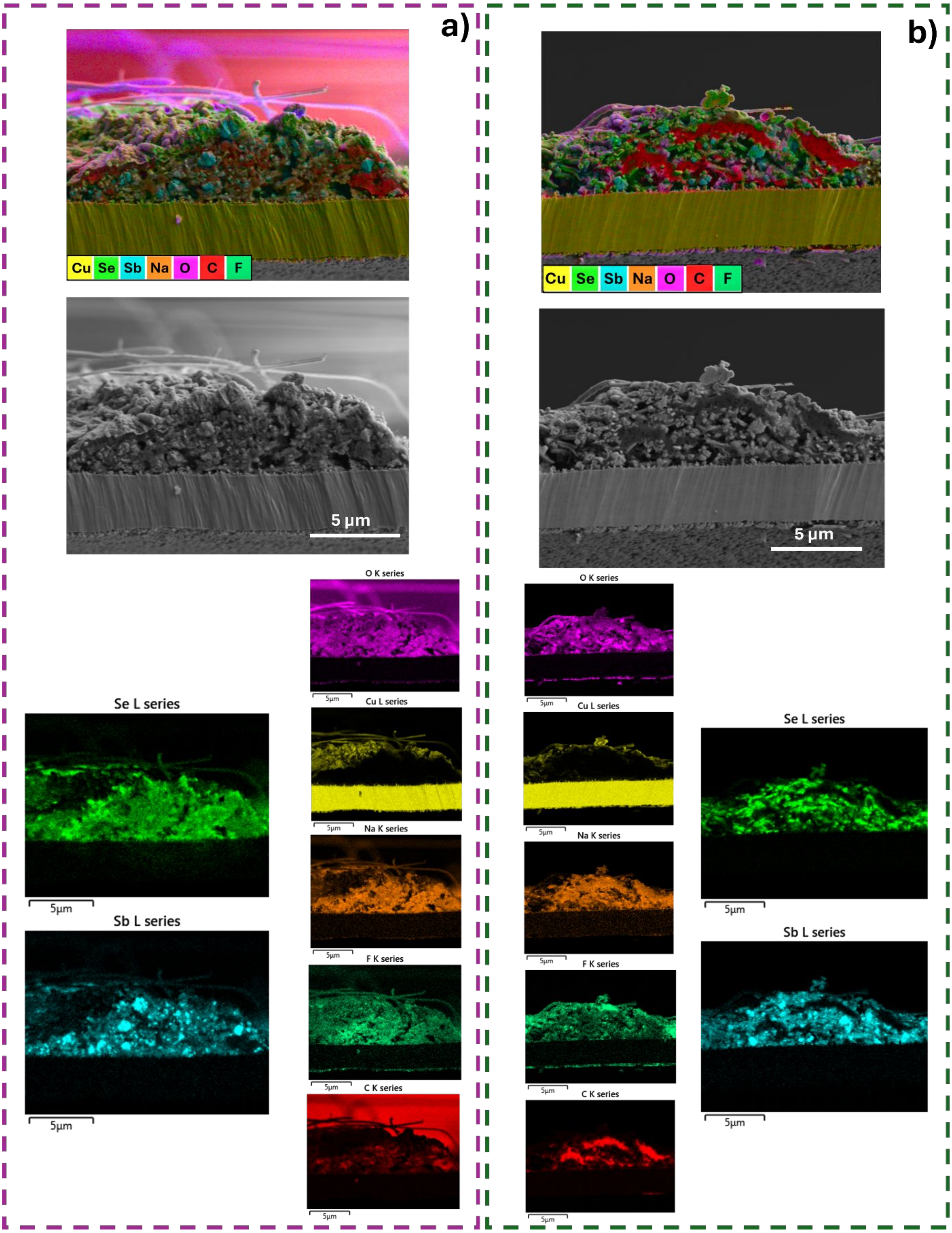

Figure S10. Cross-sectional EDX mapping of Sb_2Se_3/Sb-based electrodes retrieved after **a) (purple dashed line)** the 5th sodiation, and **b) (green dashed line)** the 5th de-sodiation. The EDX mapping of the 5th sodiation in **a)** showed that the whole electrode was sodiated (orange color in map) and that phases corresponding to Na_3Sb (green color) and Na_2Se (cyan color) and Na_5SbSe (cyan and green colors) could be observed. The EDX mapping of the 5th de-sodiation in **b)** showed that elemental Sb (cyan color) is formed (2.00 V) and Na_2Se remained a stable compound (green color).

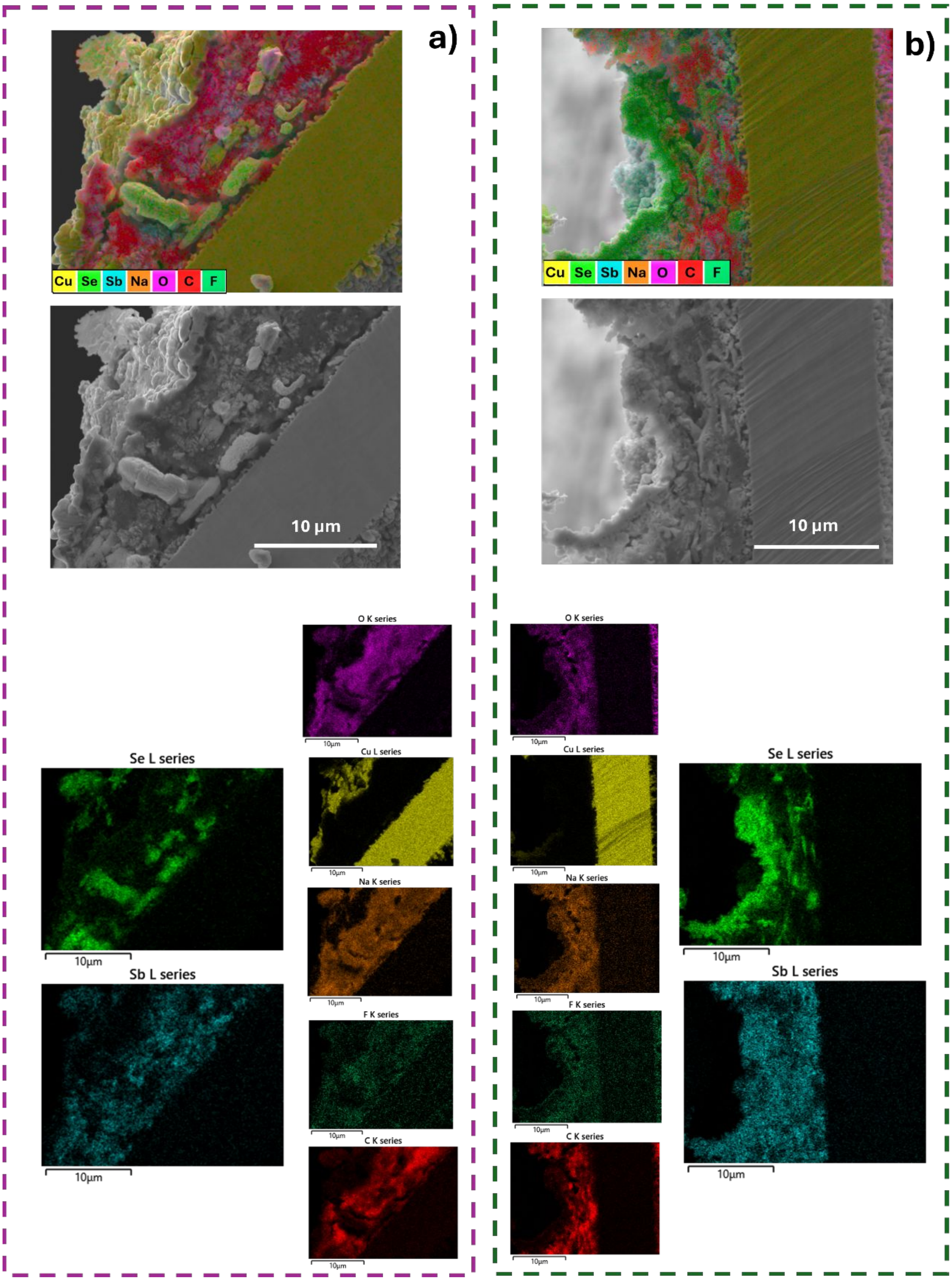

Figure S11. Cross-sectional EDX mapping of Sb_2Se_3/Sb-based electrodes retrieved after **a) (purple dashed line)** the 10th sodiation, and **b) (green dashed line)** the 10th de-sodiation. The EDX mapping of the tenth sodiation in **a)** showed that the whole electrode was sodiated (orange color in map) and that phases corresponding to Na_3Sb (cyan color) and Na_2Se (green color) and Na_5SbSe (cyan and green colors) could be observed. Cu (yellow color) had reacted in the ion-mill and was deposited on the surface of the electrode. The EDX mapping of the 10th de-sodiation in **b)** showed that elemental Sb (cyan color) is formed (2.00 V) and Na_2Se remained a stable compound (green color).

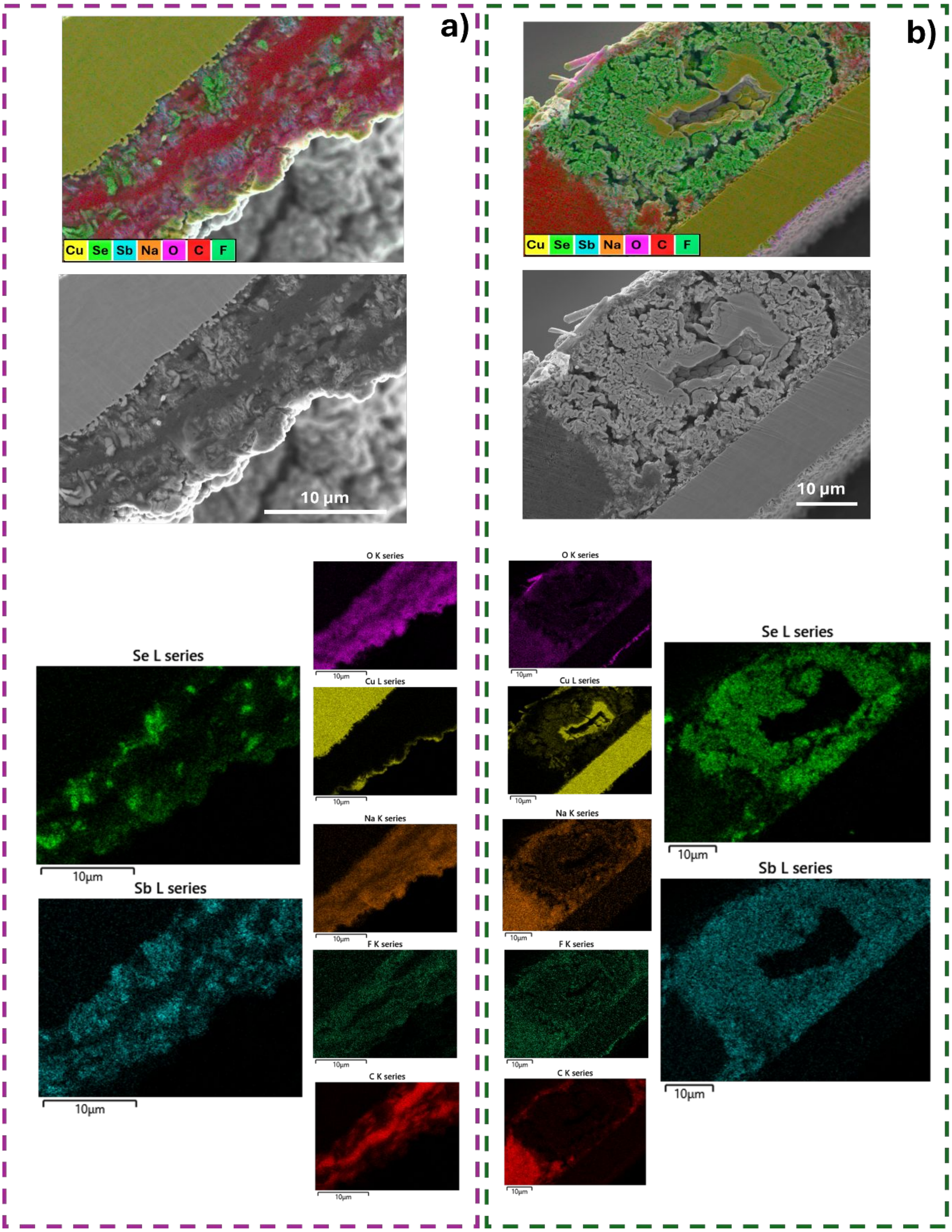

Figure S12. Cross-sectional EDX mapping of Sb_2Se_3/Sb-based electrodes retrieved after **a) (purple dashed line)** the 20th sodiation, and **b) (green dashed line)** the 20th de-sodiation. The EDX mapping of the 20th sodiation in **a)** showed that the whole electrode was sodiated (orange color in map) and that phases corresponding to Na_3Sb (cyan color) and Na_2Se (green color) and Na_5SbSe (cyan and green colors) could be observed. The EDX mapping of the 20th de-sodiation in **b)** showed that elemental Sb (cyan color) is formed (2.00 V) and Na_2Se remained a stable compound (green color).

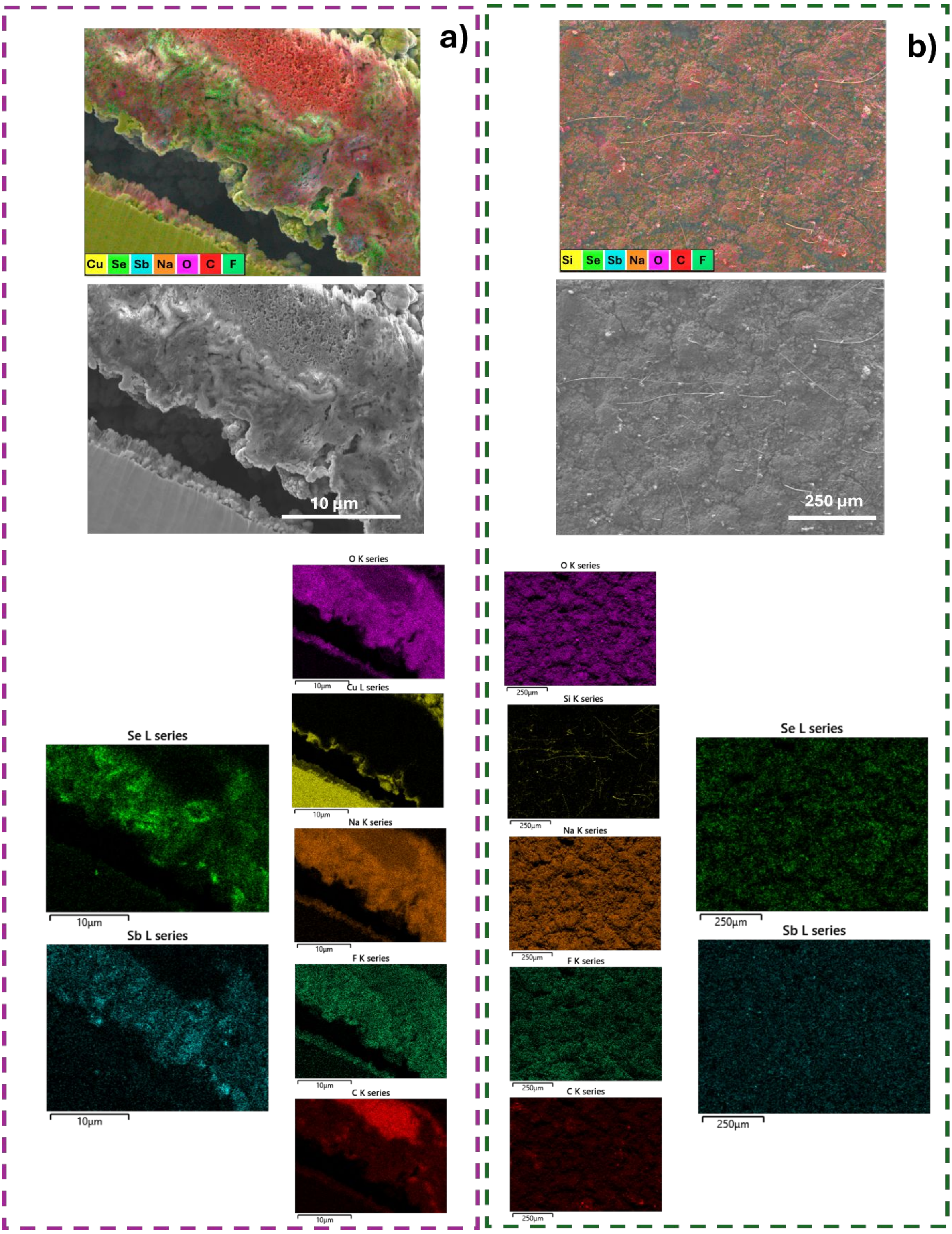

Figure S13. a) (dashed purple line) Cross-sectional EDX mapping of Sb_2Se_3/Sb-based electrode retrieved after the 120th sodiation. **b) (dashed green line)** EDX mapping of the surface of the same Sb_2Se_3/Sb-based electrode retrieved after the 120th sodiation. The EDX maps indicated that Na is homogeneously dispersed throughout the electrode coating (orange color in map), but that the coating had delaminated from the Cu current collector (yellow color in map) visible in **a)**.

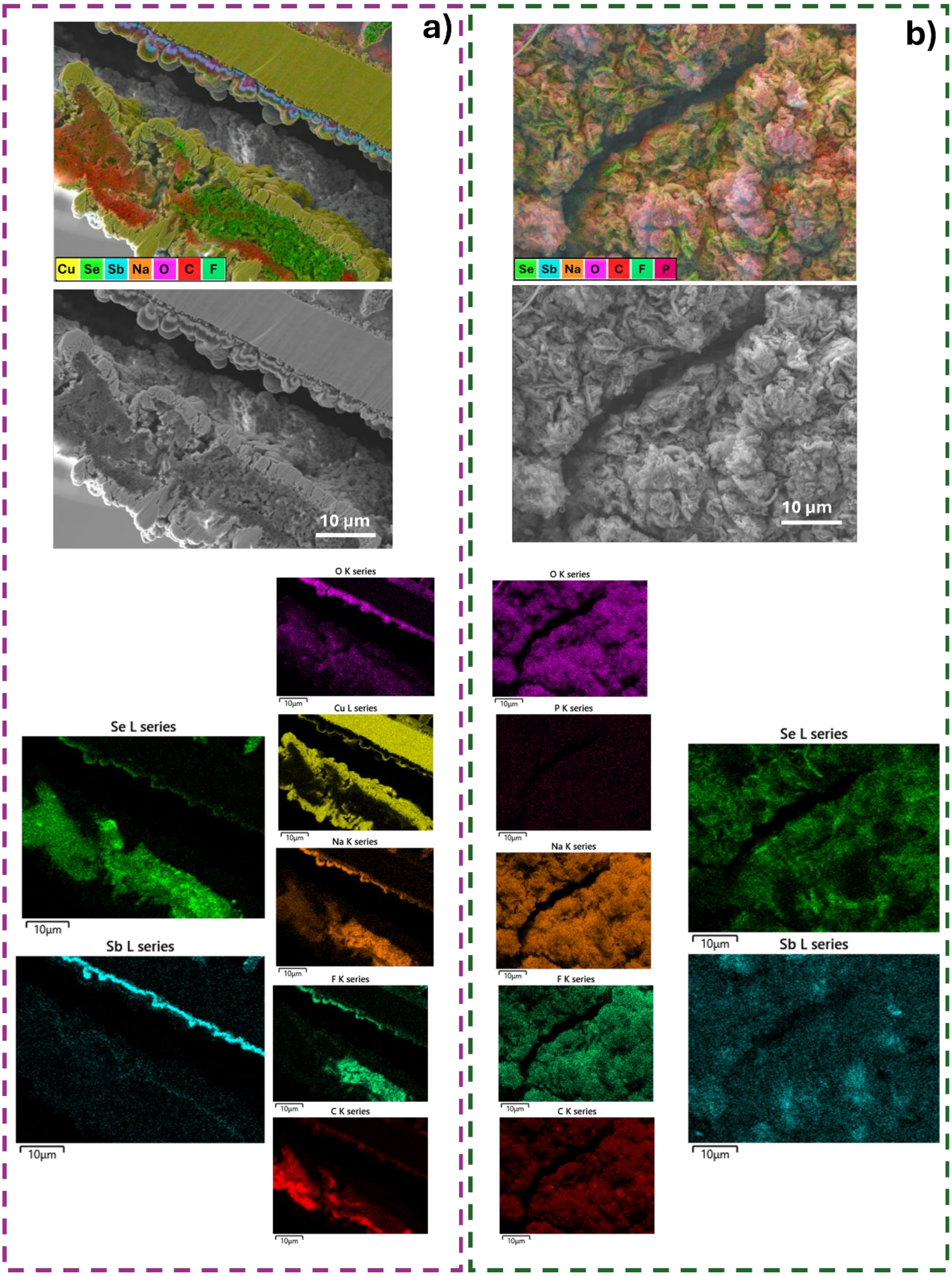

Figure S14. a) (dashed purple line) Cross-sectional EDX mapping of Sb_2Se_3/Sb-based electrode retrieved after the 120th de-sodiation. **b) (dashed green line)** EDX mapping of the surface of the same Sb_2Se_3/Sb-based electrode retrieved after the 120th de-sodiation. The EDX maps indicated that the Na is homogeneously dispersed throughout the electrode coating (orange color in map), but that the coating had delaminated from the

Cu current collector (yellow color in map) visible in **a)**. Cu (yellow color in map) had deposited on top of the coating during ion-milling.

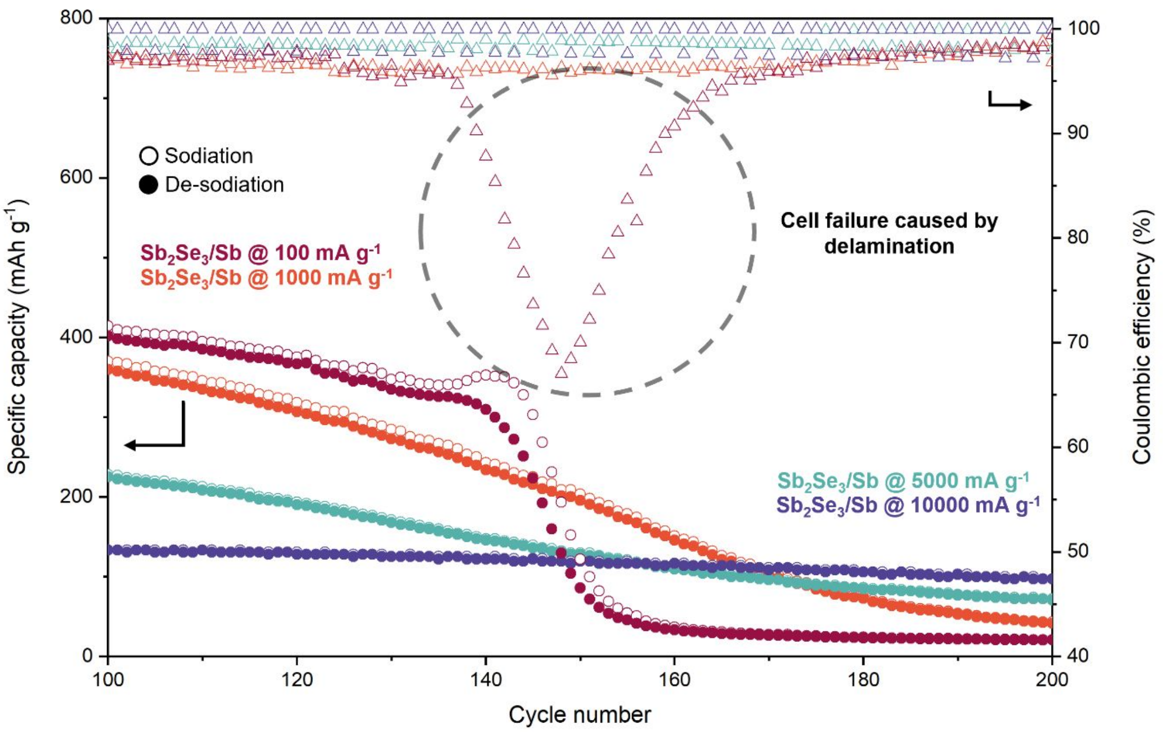

Figure S15. Specific capacity per cycle plots of Sb_2Se_3/Sb composite cycled at 100 mA g^{-1}, 1000 mA g^{-1}, 5000 mA g^{-1} and 10000 mA g^{-1} cycled in Na half-cells between 0.01 V and 2.00 V. The curves are extracted from the same cycling data of cells displayed in Figure 1 in Main (here shown between cycle 100 and 200). Dashed grey circle indicate the rapid fade in CE caused by delamination of the electrode active material component from the current collector, which was revealed by SEM-EDX analysis at the 120th de-sodiation cycle in Figure 3 and S11-12.

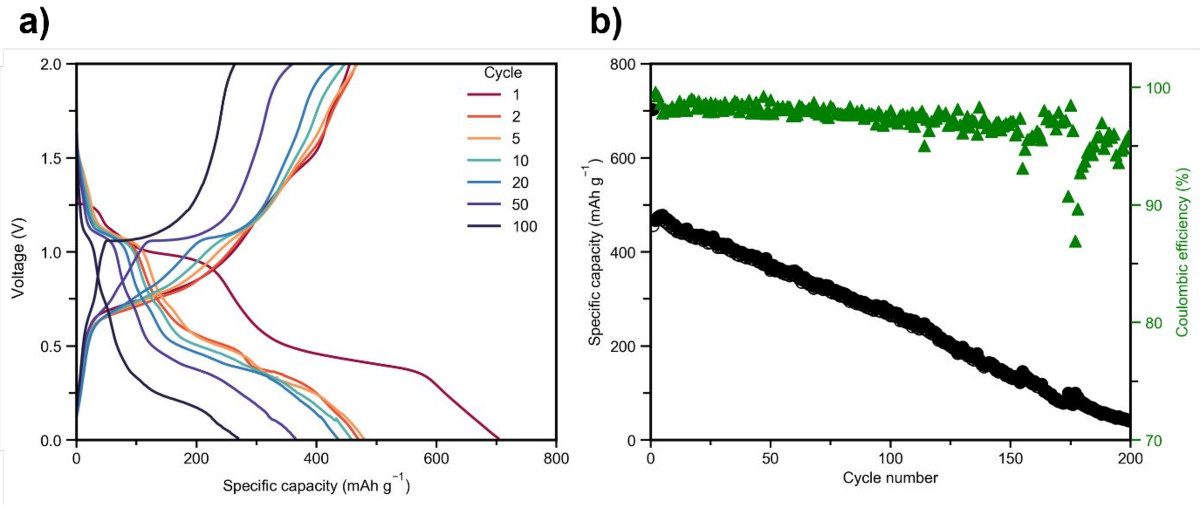

Figure S16. a) Galvanostatic sodiation/de-sodiation curves of Sb_2Se_3/Sb coated on carbon coated Al foil in Na-half cell cycled between 0.01 V and 2.00 V *vs.* Na/Na^+ using a specific current density of 100 mA g^{-1}. **b)** Specific capacity upon cycling and Coulombic efficiency trend.

References

(1) Ou, X.; Yang, C.; Xiong, X.; Zheng, F.; Pan, Q.; Jin, C.; Liu, M.; Huang, K. A New rGO-Overcoated Sb2Se3 Nanorods Anode for Na+ Battery: In Situ X-Ray Diffraction Study on a Live Sodiation/Desodiation Process. *Advanced Functional Materials* **2017**, *27*, 1606242. DOI: 10.1002/adfm.201606242.

(2) Klayman, D. L.; Griffin, T. S. Reaction of selenium with sodium borohydride in protic solvents. A Facile Method for the introduction of selenium into organic molecules. *Journal of the American Chemical Society* **1973**, *95*, 197-199. DOI: 10.1021/ja00782a034.

(3) Skurtveit, A.; Pastusic Jr., A.; Brennhagen, A.; Maddar, F. M.; Mohn, C. E.; Karmakar, A.; O'Keefe, C. A.; Hasa, I.; Cavallo, C.; Arstad, B.; et al. Unique Na5-xSbSe phase enables high-rate performance of Sb2Se3 anodes in Na-ion batteries. *Advanced Energy Materials* **2025**. DOI: 10.1002/aenm.202501433.

(4) Drozhzhin, O. A.; Tereshchenko, I. V.; Emerich, H.; Antipov, E. V.; Abakumov, A. M.; Chernyshov, D. An electrochemical cell with sapphire windows for operando synchrotron X-ray powder diffraction and spectroscopy studies of high-power and high-voltage electrodes for metal-ion batteries. *Journal of Synchrotron Radiation* **2018**, *25*, 468-472. DOI: 10.1107/S1600577517017489.

(5) Dyadkin, V.; Pattison, P.; Dmitriev, V.; Chernyshov, D. A new multipurpose diffractometer PILATUS@SNBL. *Journal of Synchrotron Radiation* **2016**, *23*, 825-829. DOI: 10.1107/S1600577516002411.

(6) Marshall, K. *X-ray-detector-gain-map*. 2025. https://github.com/msujas/X-ray-detector-gain-map (accessed: February 2025).

(7) Skurtveit, A. *OPAL-XRD*. 2025. https://github.com/piieceofcake/OPAL-XRD (accessed: February 2025).

(8) Lu, X.; Wang, Z.; Liu, K.; Luo, J.; Wang, P.; Niu, C.; Wang, H.; Li, W. Hierarchical Sb2MoO6 microspheres for high-performance sodium-ion battery anode. *Energy Storage Materials* **2019**, *17*, 101-110. DOI: 10.1016/j.ensm.2018.11.021.

(9) Cui, J.; Zheng, H.; Zhang, Z.; Hwang, S.; Yang, X.-Q.; He, K. Origin of anomalous high-rate Na-ion electrochemistry in layered bismuth telluride anodes. *Matter* **2021**, *4*, 1335-1351. DOI: 10.1016/j.matt.2021.01.005.

(10) Kravchyk, K. V.; Kovalenko, M. V.; Bodnarchuk, M. I. Colloidal Antimony Sulfide Nanoparticles as a High-Performance Anode Material for Li-ion and Na-ion Batteries. *Scientific Reports* **2020**, *10*, 2554. DOI: 10.1038/s41598-020-59512-3.
